# Supplementary material for: Polyphenols Attenuate Highly-Glycosylated Haemoglobin-Induced Damage in Human Peritoneal Mesothelial Cells
Source: Antioxidants (Basel). 2020 Jul 1;9(7):572. doi: 10.3390/antiox9070572 (PMC7402166; doi:10.3390/antiox9070572)
Supplement: Supplementary file 1 [file antioxidants-09-00572-s001.pdf]

Table S1. Characteristics of the patients, mean age 54.66 ±5.08; range 23-79 yrs.*Patients with a diagnosis of cancer were free of metastatic disease and peritoneal involvement.

**Age**	**Gender**	**Diagnosis***	**Drugs**
21	F	Colonic cancer	No
23	M	Hepatic hydatidosis	No
27	M	Rectal cancer	No
32	F	Rectal cancer	No
39	F	Cholelitiasis	Omeprazol Lorazepam<br>Fluoxetin
42	F	Rectal cancer	No
44	F	Rectal cancer	No
46	M	Colonic cancer	No
51	M	Colonic cancer	No
55	M	Colonic cancer	No

Cell culture

HPMCs were isolated from omental tissue from 10 different donors (free of any cardiovascular or peritoneal disease and non-taking anti-inflammatory drugs or antioxidants) undergoing nonurgent, non-septic abdominal surgery, using previously described methods (Chung-Welch et al., 1997). HPMCs were routinely cultured in M199 containing 1 g l^{-1} of D-glucose and supplemented with 10% FCS, 100 $mgml^{-1}$ streptomycin, 100Uml^{-1} penicillin, and 2.5 $mgml^{-1}$ amphotericin. At confluence, HPMC were passaged using a 0.02% EDTA–0.05% trypsin solution and split in a 1:2 ratio.

HPMCs characterization was based on both cell morphology (immediately prior to and at confluence, cells adopted the polygonal cobblestone-like appearance characteristic of epithelial cells and formed a monolayer) and indirect immunofluorescence staining of several human mesothelial markers (Chung-Welch et al., 1997). In brief, HPMCs showed a diffuse positive staining with an anti-von Willebrand factor antibody (Dakopatts, Glostrup, Denmark) and a marked staining with anti-cytokeratins 8 and 18, anti-E-cadherin, and anti-vimentin antibodies (all of them from Sigma Chemical Co.). HPMCs failed to express the endothelial marker PECAM-1 (CD31) (see Supplemental data, Table 2).

Table S2. HPMC characterization.

Markers	*HPMC*	*HAEC*	*HUVEC*	*HASMC*
Factor VIII (vWF)	+/-<br>(diffuse)	+++<br>(granular)	+++<br>(granular)	negative
α-actin	negative	negative	negative	+++
E-Cadherin	++	negative	negative	
VE-Cadherin	+++	++	++	
Vimentin	+++	++	++	
Cytokeratin 8	++	++	++	
Cytokeratin 18	++	++	++	
PECAM-1(CD31)	negative	++		

HPMC, Human Peritoneal Mesothelial Cells; **HAEC**, Human Aortic Endothelial Cells; **HUVEC**, Human Umbilical Vascular Endothelial Cells; **HASMC**, Human Aortic Smooth Muscle Cells. The morphologic and immunofluorescence-staining features of the

cells remained stable throughout the passages used.

Preparation of Amadori adducts

Lyophilised human haemoglobins, nonenzymatically glycosylated at either elevated or normal levels, containing 11.1% and 5.4% HbA1, respectively, were purchased from Sigma. Briefly, haemoglobins were dissolved in deionised water and subsequently reduced by incubation with an excess of sodium dithionite. The haemoglobin solutions were then extensively dialysed using a 0.25A˚ pore diameter (approximately 12 kDa mol wt) dialysis membrane (Viskings, Serva, Heidelberg, Germany) against deionised water containing $10mgl^{-1}$ EDTA and continuously bubbled with N2. Oxyhaemoglobins were then aliquoted and stored at -70 ℃ until used.

The absence of AGEs in the glycated oxyhaemoglobin solutions was assessed by measuring fluorescence in a Fluostar fluorometer (BMG Labtechnologies, Offenburg, Germany) at excitation maximum of 370 nm and emission maximum of 440 nm, which allows quantifying total AGEs (Sell & Monnier, 1989). A standard curve (r¼0.99) was carried out using AGE-modified BSA (0.5–5 $mgml^{-1}$), prepared following a previously described method (Bucala et al., 1991). Glycated preparations did not contain significative bacterial endotoxin contamination (p0.5U endotoxin ml^{-1}), as measured with Pyrogents plus kit (Biowhittaker Europe SPRL, Verners, Belgium).
